# Time-Specific Fear Acts as a Non-Photic Entraining Stimulus of Circadian Rhythms in Rats

**DOI:** 10.1038/srep14916

**Published:** 2015-10-15

**Authors:** Blake A. Pellman, Earnest Kim, Melissa Reilly, James Kashima, Oleksiy Motch, Horacio O. de la Iglesia, Jeansok J. Kim

**Affiliations:** 1 Department of Psychology, University of Washington, Seattle, Washington 98195, USA.; 2 Department of Biology, University of Washington, Seattle, Washington 98195, USA.; 3 Program in Neuroscience, University of Washington, Seattle, Washington 98195, USA

## Abstract

Virtually all animals have endogenous clock mechanisms that “entrain” to the light-dark (LD) cycle and synchronize psychophysiological functions to optimal times for exploring resources and avoiding dangers in the environment. Such circadian rhythms are vital to human mental health, but it is unknown whether circadian rhythms “entrained” to the LD cycle can be overridden by entrainment to daily recurring threats. We show that unsignaled nocturnal footshock caused rats living in an “ethological” apparatus to switch their natural foraging behavior from the dark to the light phase and that this switch was maintained as a free-running circadian rhythm upon removal of light cues and footshocks. Furthermore, this fear-entrained circadian behavior was dependent on an intact amygdala and suprachiasmatic nucleus. Thus, time-specific fear can act as a non-photic entraining stimulus for the circadian system, and limbic centers encoding aversive information are likely part of the circadian oscillator network that temporally organizes behavior.

Most animals exhibit rhythmic patterns of activity that are restricted to specific periods of the daily cycle, such as the daytime (diurnal), nighttime (nocturnal), or dawn-and-dusk times (crepuscular)^1^,^2^,^3^. Such circadian rhythms are generated by endogenous molecular clocks that oscillate with approximately 24-h periods, but because these periods are usually slightly shorter or longer within individuals, these clocks must be “entrained” by external cues (*zeitgebers* or “time-givers”) to remain environmentally relevant^1^,^2^,^3^,^4^,^5^. These functions are thought to be critical in crafting an ecological niche by coordinating psychophysiological functions to balance optimal times for exploring resources (e.g., food, water, and mates) and avoiding predatory threats in the environment^6^,^7^,^8^,^9^,^10^,^11^.

The principal zeitgeber is an organism’s local light-dark (LD) cycle^2^,^3^,^4^,^5^. Via projections from light-sensitive retinal ganglion cells, the LD cycle entrains the “master” circadian clock located within the suprachiasmatic nucleus (SCN) in mammals^1^,^2^,^12^,^13^,^14^. This master clock then drives secondary “slave” clocks in other brain regions or peripheral organs to coordinate daily physiological and behavioral rhythms^14^,^15^,^16^. Cyclic stimuli other than light (non-photic cues), such as ambient temperature and time-restricted feeding schedules, have also been found to entrain circadian rhythms^17^,^18^,^19^,^20^.

In humans, circadian rhythms are vital to mental health as they are often disturbed in psychopathologies^21^,^22^,^23^. While clinical and experimental studies have shown that emotional states, such as fear, anxiety, and depression, can disrupt circadian rhythms^21^,^22^,^23^,^24^,^25^,^26^, it remains unknown whether they can serve entraining functions. Fear is a crucial, highly-conserved mechanism of survival that guides behaviors that help organisms minimize exposure to threats in their habitat^8^,^27^,^28^,^29^,^30^,^31^, and it is conceivable that cyclic daily threats may act as entraining environmental stimuli to the circadian system.

The present study investigated the significance of emotions, specifically fear, on circadian rhythms in rats under naturalistic conditions where defensive and appetitive behaviors were all a meaningful, integrated part of the animals’ lives. Rats lived for extended periods in “closed economy” chambers^32^,^33^,^34^,^35^, comprised of a safe, bedded nest and a risky foraging area that had to be entered to obtain food and water (Fig. 1a). The foraging zone was rendered dangerous by administering either signaled or unsignaled footshocks only during the dark phase of the LD cycle (Fig. 1b), which is typically the natural active phase for rats. A closed economy paradigm was chosen to allow experimental animals to have control over their own appetitive and defensive behaviors and reflect a more natural foraging situation. In response to unsignaled nocturnal shock, animals switched their natural feeding and activity from the dark phase to the light phase. This fear-induced diurnal behavior persisted (free-ran) when the light cues and footshocks were removed (Fig. 1c), and the phase of the free-running rhythm approximated the phase when the recurring threat was present, confirming that daily cyclic fear can act as a zeitgeber. Additionally, the expression of this fear-entrained circadian rhythm was dependent on an intact amygdala and SCN. The finding that amygala-coded fear can reprogram SCN-directed circadian behavior suggests that the amygdala is a part of the circadian oscillator network that temporally organizes behavior.

## Results

Animals living in the closed economy chamber and maintained on a 12-h/12-h LD cycle quickly learned to press a lever to procure food pellets (a continuous reinforcement schedule) in the foraging area. As expected, rats preferred to forage during the dark phase during baseline (7 d), as measured by feeding and total locomotion [mixed-model ANOVA, see Methods; pellets: *F*_1, 14_ = 112.69, *p* < 0.001; activity: *F*_1, 14_ = 81.51, *p* < 0.001] (Fig. 2). When exposed to the risk of unsignaled nocturnal shocks, rats increased their locomotor and feeding behavior during the light phase and decreased locomotor and feeding behavior during the dark phase [phase × day: pellets, *F*_13, 182_ = 9.03, *p* < 0.001; activity, *F*_13, 182_ = 11.87, *p* < 0.001] (Fig. 2). Although rats remained more active during the dark phase than the light phase overall [*F*_1, 14_ = 45.68, *p* < 0.001], they preferred to obtain food during the light phase by the end of the unsignaled shock period [last 2 d, L vs. D: dark: 203.44 ± 24.58 pellets; light: 335.41 ± 34.15 pellets; *t*_15_ = 2.60, *p* = 0.02]. The effects of signaled nocturnal shock varied depending on the order in which it was experienced. For the rats that experienced unsignaled shock first (Fig. 2a–d), diurnal behavior persisted into the signaled footshock period because the avoidance of footshocks (i.e., not foraging during the dark phase) served as negative reinforcement^36^ [phase × day × order: feeding, *F*_4.055, 56.776_ = 3.05, *p* = 0.023; activity, *F*_4.865, 68.108_ = 2.50, *p* = 0.040; *d.f.* adjusted]. In contrast, the rats that were exposed to the risk of signaled nocturnal footshocks at the outset (Fig. 2e–h) quickly learned to avoid the footshock and thereby maintained their baseline circadian behavior, only to become diurnal when subsequently switched to unsignaled nocturnal footshock condition (Fig. 3). Thus, the rats that had been exposed to unsignaled shock first were significantly less active [114.26 ± 11.65 m; *t*_14_ = 2.68, *p* = 0.018] and ate fewer pellets [295.80 ± 35.67; *t*_14_ = 5.10, *p* < 0.001] in the dark phase and were significantly more active [77.01 ± 5.37 m; *t*_14_ = 4.29, *p* < 0.001] and ate more pellets [325.74 ± 34.41; *t*_10.44_ = 3.86, *p* = 0.003; *d.f.* adjusted] in the light phase compared to the rats that experienced signaled shock first (dark: 150.69 ± 7.04 m, 502.29 ± 19.12 pellets; light: 46.91 ± 4.53 m, 176.65 ± 17.64 pellets).

It is interesting to note that during the unsignaled nocturnal shock condition, animals began to increase their activity and feeding before the switch from the dark to the light phase (Fig. 4; red lines). To analyze this behavior, we compared the mean activity and feeding behavior of the rats across the last 5 d of baseline and the unsignaled shock condition in 10-min time bins during the last 4 h of the dark phase (Zeitgeber Time (ZT) 20–24/0, with ZT0/24 being the time of lights on and ZT12 being the time of lights off; see Methods). Feeding behavior (Fig. 4a; left side) and activity (Fig. 4a; right side) significantly increased before the onset of the light phase (ZT0) in the unsignaled nocturnal shock condition relative to baseline [condition × time: activity, *F*_23, 92_ = 2.85, *p* < 0.001; feeding, *F*_23, 92_ = 3.35, *p* < 0.001]. This *anticipatory* behavior is incompatible with the possibility that the light simply served as a safety cue for the animals to start foraging (i.e., not a Pavlovian response). In contrast, animals experiencing signaled nocturnal shock after baseline did not show a significant increase in feeding or activity during this time [condition × time: activity, *F*_23, 92_ = 0.95, *p* = 0.543; feeding, *F*_23, 92_ = 1.48, *p* = 0.099] (Fig. 4b) relative to baseline.

**Figure 1:**
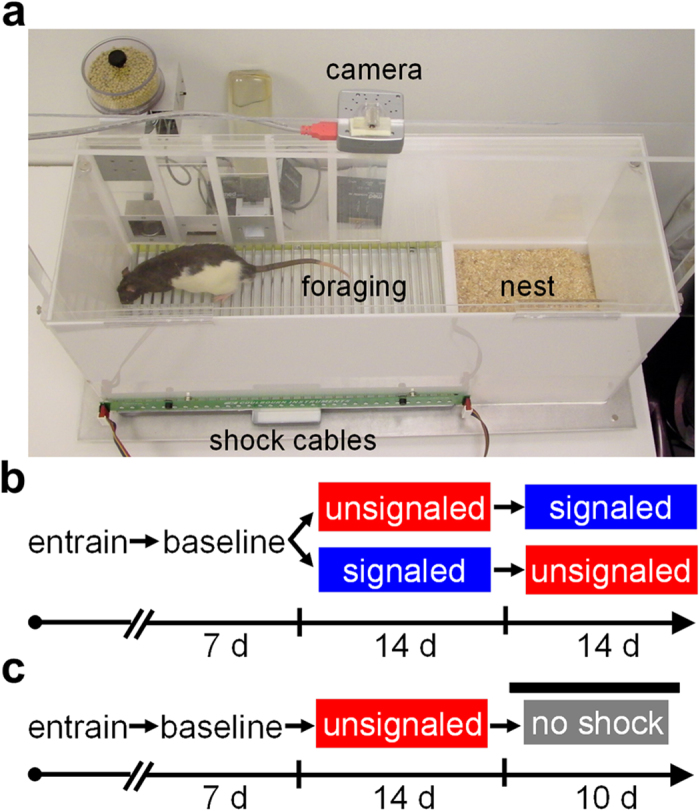
Experimental apparatus and design. (**a**) Photograph of the closed economy apparatus. Diagram of the experimental designs for (**b**) the primary experiment and (**c**) the free-running experiment.

**Figure 2:**
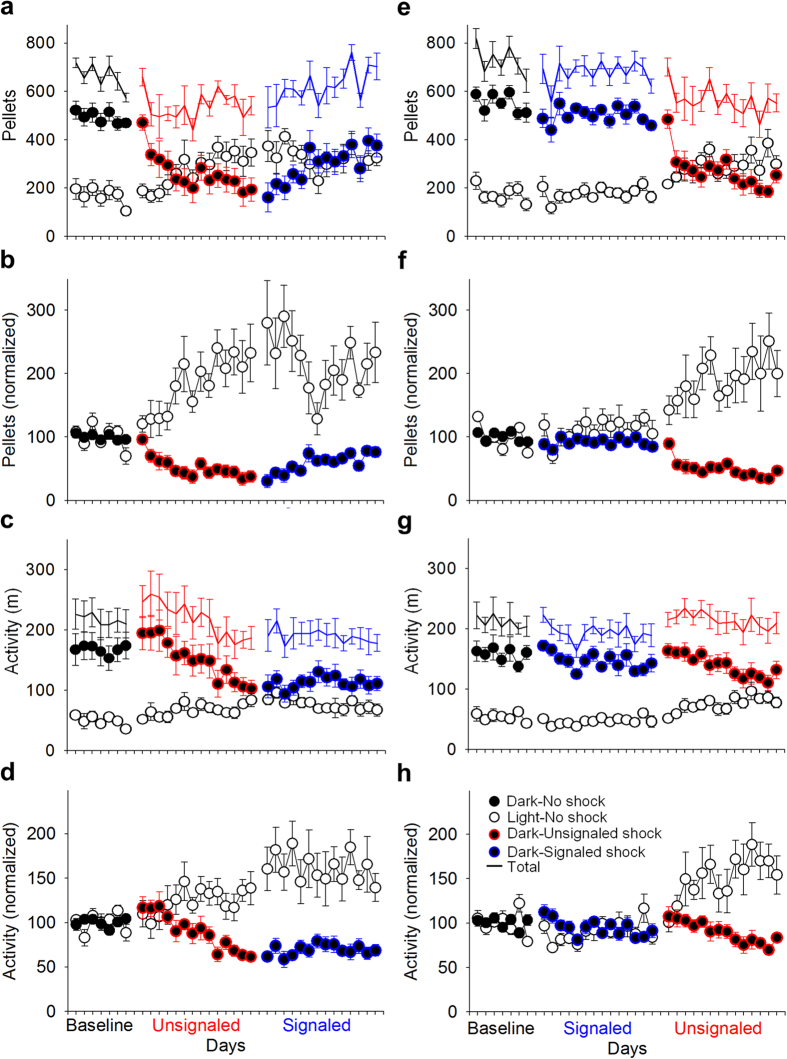
Effects of nocturnal shock on foraging and activity patterns. (**a**) Raw number of pellets obtained, (**b**) number of pellets obtained normalized to baseline (*black*) average, (**c**) raw activity (distance traveled in m), and (**d**) activity normalized to baseline average of rats that experienced unsignaled (*red*) nocturnal footshocks before signaled (*blue*) nocturnal footshocks (n = 8). (**e**) Raw number of pellets obtained, (**f**) number of pellets obtained normalized to baseline (*black*) average, (**g**) raw activity (distance traveled in m), and (**h**) activity normalized to baseline average of rats that experienced signaled (*blue*) nocturnal footshocks before unsignaled (*red*) nocturnal footshocks (n = 8). When exposed to unsignaled shock, rats shift from natural nocturnal behaviors to diurnal behaviors. During signaled shock, behavior depended on whether unsignaled shock was already experienced or not. All error bars represent SEM.

**Figure 3:**
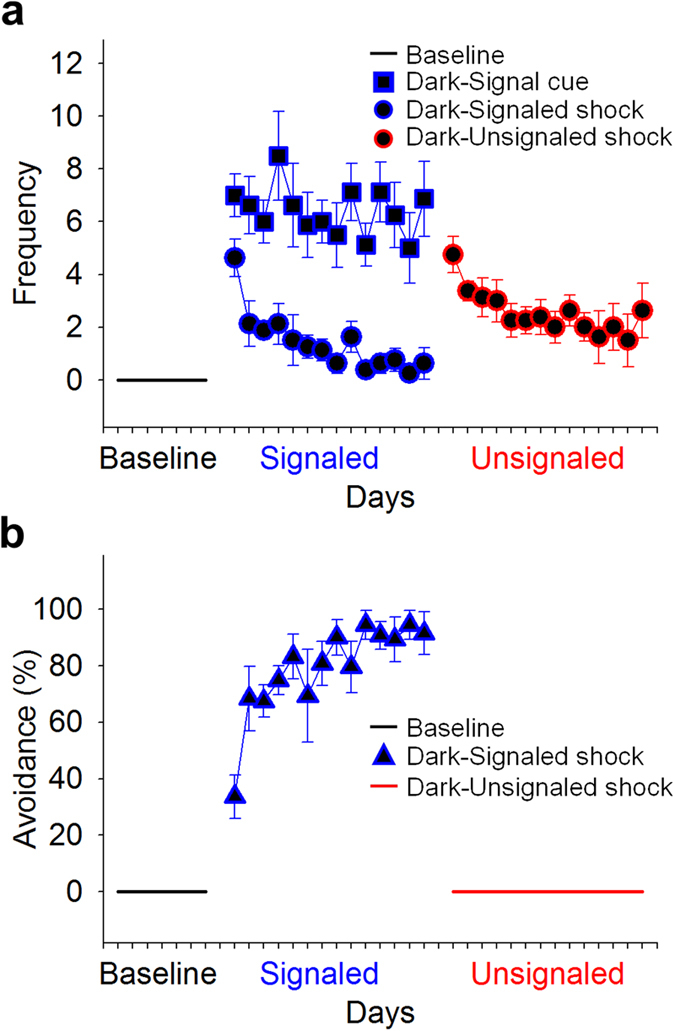
Light cue, footshock, and avoidance during nocturnal shock. (**a**) Mean number of light cue presentations (*squares*) and mean number of shocks (*circles*) animals (n = 8) received during signaled shock and unsignaled shock conditions. (**b**) Mean percentage of avoidance responses made (i.e., moving from foraging area to the nest) during the signaled shock (<9 s of 10 s light cue). All error bars indicate SEM.

**Figure 4:**
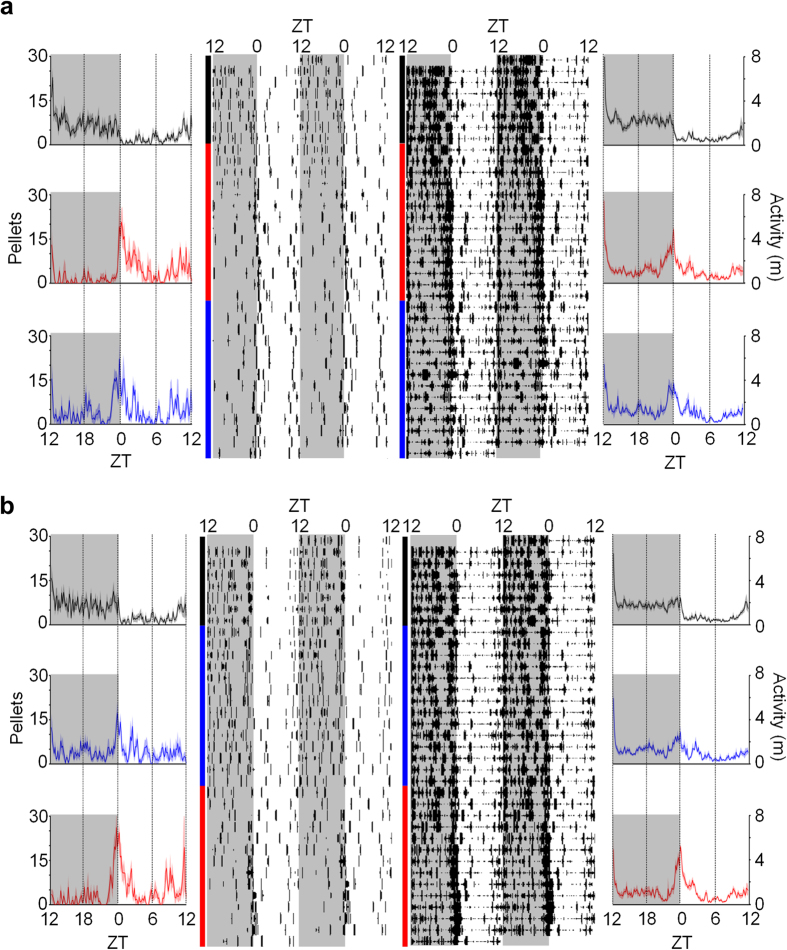
Effects of nocturnal shock on circadian rhythms. 24-h waveforms (*outside*) and raster plots (*inside*) of feeding (number of pellets obtained; *left*) and activity (distanced traveled in m; *right*) through baseline (*black*), unsignaled (*red*) and signaled shock (*blue*) conditions in rats that (**a**) experienced unsignaled shock first (n = 8) and (**b**) experienced signaled shock first (n = 8). Waveforms show mean feeding/activity over 24 h (bold lines), in 10-min time-bins, averaged over the last 5 d of each condition. Raster plots are from a representative animal from each group. Gray shaded areas (ZT12-24/0) indicate dark/shock phase. Behavior during nocturnal shock conditions increases 4 h leading up to the dark-to-light transition (ZT0). SEM is represented by shaded areas above and below the bold lines.

**Figure 5:**
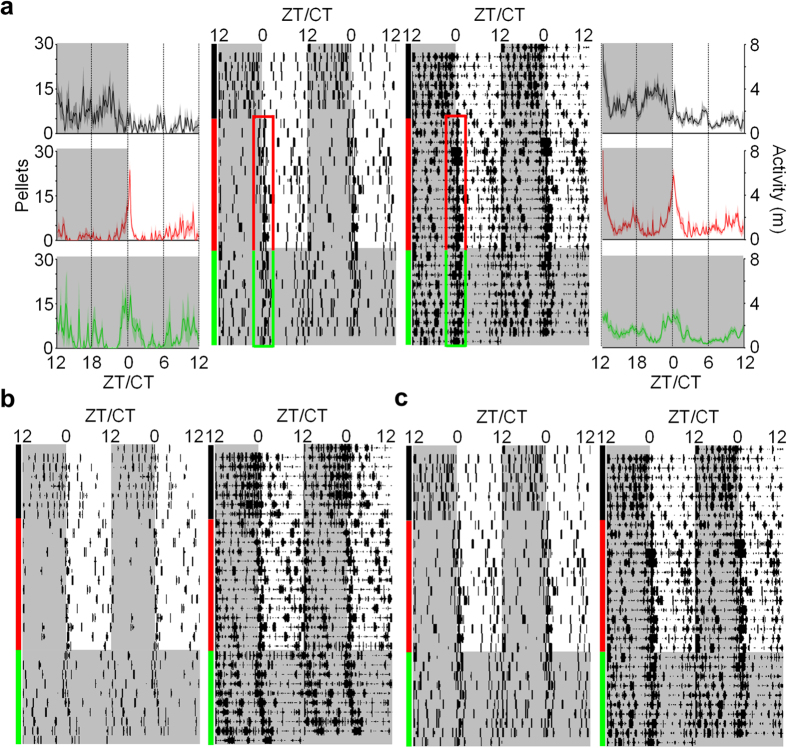
Fear-entrained anticipatory circadian feeding and activity. (**a**) 24-h waveforms and raster plots of feeding (number of pellets obtained; *left*) and activity (distanced traveled in m; *right*) through baseline (*black*), unsignaled shock (*red*), and constant dark conditions (*green*) demonstrate fear-induced, anticipatory circadian rhythms. Waveforms show mean (n = 5) feeding/activity over 24 h (bold lines) averaged over the last 5 d of each condition. Mean waveforms of feeding (*bottom left;* n = 3) and activity (*bottom right;* n = 5) under constant dark conditions demonstrate free-running rhythms. Phases are aligned according to free-running onset of feeding or activity respectively. SEM is shown as the shaded areas above and below the bold lines. The raster plots are from a representative animal. The red outline highlights the anticipatory circadian rhythm generated under unsignaled nocturnal shock conditions, which continues as a free-running rhythm (*green outline*) under constant dark conditions. Raster plots of feeding (*left*) and activity (*right*) from representative animals with free-running periods that were (**b**) greater than 24 h and (**c**) less than 24 h. Gray shaded areas represent the dark phase. Circadian times (CT) are extrapolated from the LD cycle, with CT12 being the extrapolated time of lights off (ZT12).

To test the hypothesis that fear produced by the unsignaled nocturnal shock can reprogram circadian rhythms, a group of rats was entrained and exposed to the same conditions as in the first experiment, except that after the 14 d of unsignaled nocturnal shock they underwent 10 d of constant darkness conditions without shock (Fig. 1c; see Methods). If the observed rhythmic anticipatory and diurnal foraging behavior is indeed a function of an endogenous circadian oscillator, then it should persist even when all external cyclic stimuli are removed from the environment^13^. These rats continued to display the same time-restricted feeding [waveform: *F*_95, 192_ = 1.51, *p* = 0.0084] and activity [waveform: *F*_95, 384_ = 4.78, *p* < 0.0001] throughout the free-running portion of the experiment, with a free-running phase that could be extrapolated from the phase before the release into constant conditions (Fig. 5), indicating that changes in the timing of foraging behavior are sustained by an endogenous circadian clock that is entrained by the nocturnal presentation of footshocks.

**Figure 6:**
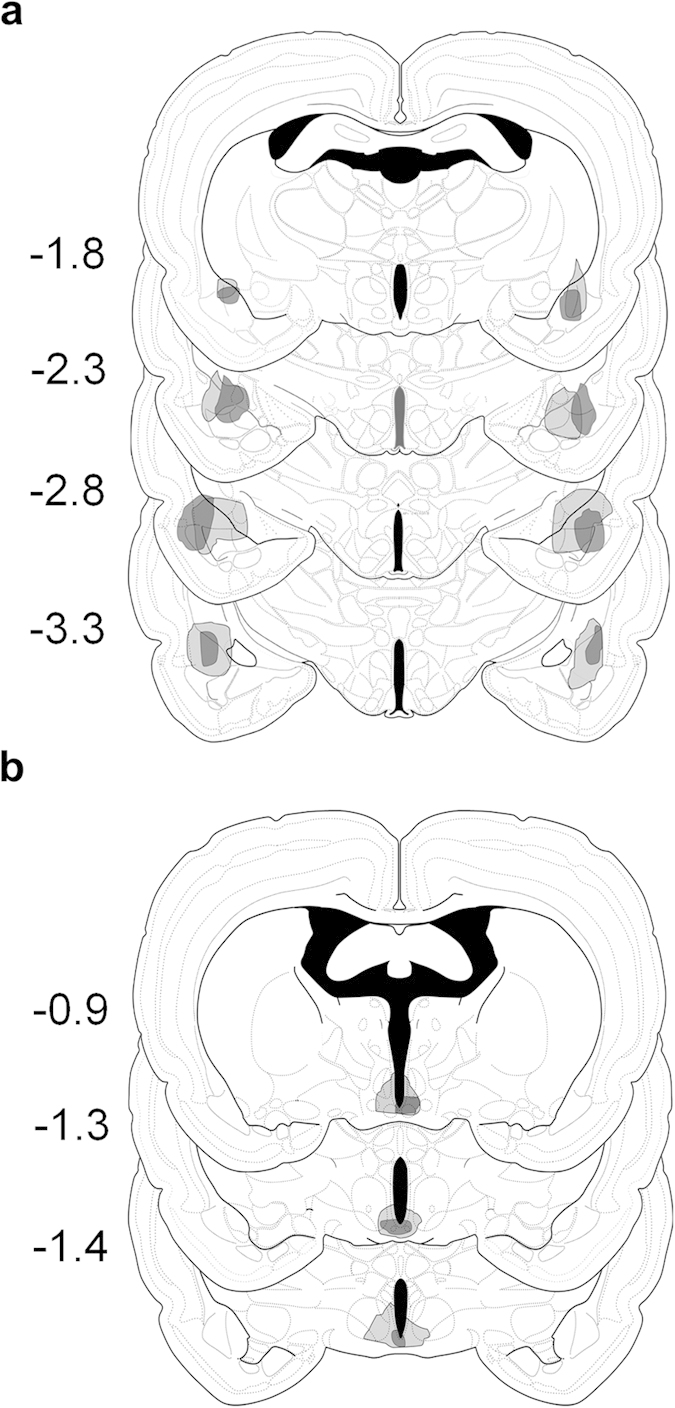
Amygdala and SCN lesions. Histological reconstruction of the smallest (dark-shaded) and largest (light-shaded) lesions of the (**a**) amygdala and (**b**) SCN. Numbers indicate mm posterior to bregma. This image is not covered by the Creative Commons Attribution 4.0 International License. Credit: Swanson, L.W.^57^ Brain maps: structure of the rat brain, 3rd edition, which is licensed under the Creative Commons Attribution-NonCommercial 4.0 International Public License. https://creativecommons.org/licenses/by-nc/4.0/.

**Figure 7:**
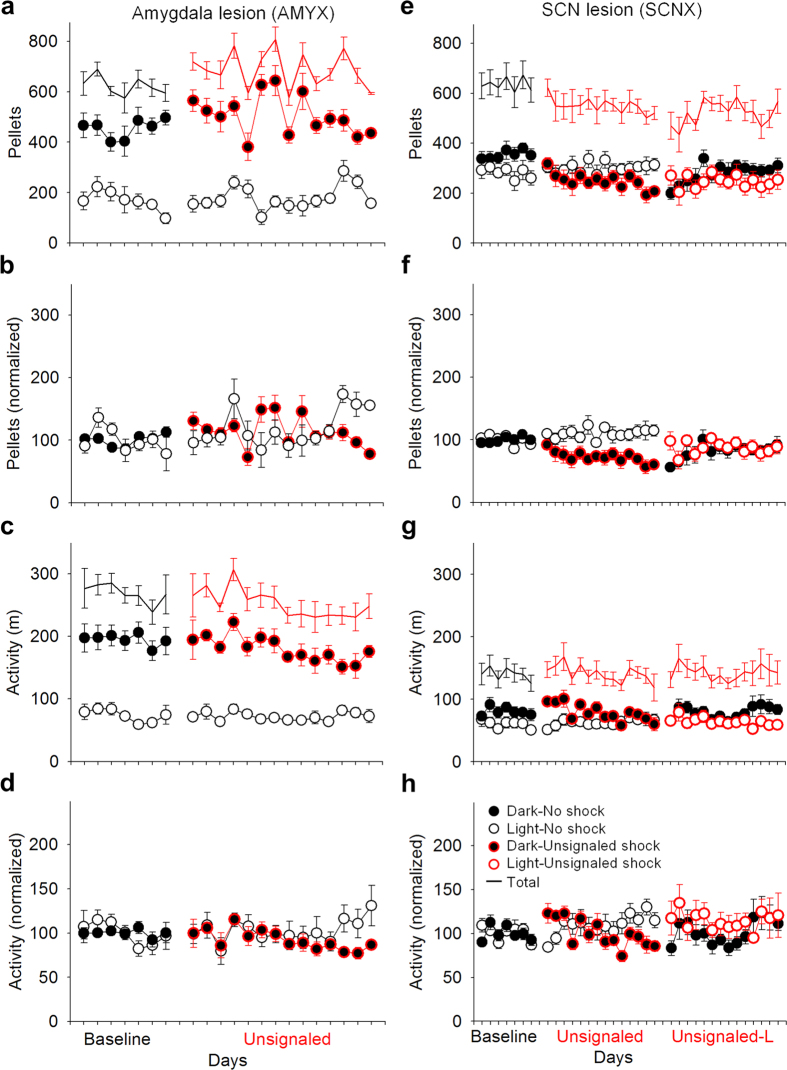
Effects of lesions on shock-induced changes to foraging and activity patterns. (**a**) Raw number of pellets obtained, (**b**) number of pellets obtained normalized to baseline average, (**c**) raw activity (distance traveled in m), and (**d**) activity normalized to baseline average of amygdala-lesioned (AMYX; n = 7) rats exposed to baseline (*black*) and unsignaled nocturnal shock (*red*) conditions. AMYX animals maintained their nocturnal feeding/activity behavior during unsignaled nocturnal shock. (**e**) Raw number of pellets obtained, (**f**) number of pellets obtained normalized to baseline (*black*) average, (**g**) raw activity (distance traveled in m), and (**h**) activity normalized to baseline average of SCN-lesioned (SCNX; n = 8) rats exposed to baseline (*black*), unsignaled nocturnal shock (*black circles with red outline*), and unsignaled diurnal shock (*open circles with red outline*) conditions. SCNX animals slightly preferred feeding in the dark phase during baseline, which abolished during unsignaled nocturnal shock. When exposed to unsignaled diurnal shock, SCNX rats’ feeding behavior remained arrhythmic. Error bars indicate SEM.

**Figure 8:**
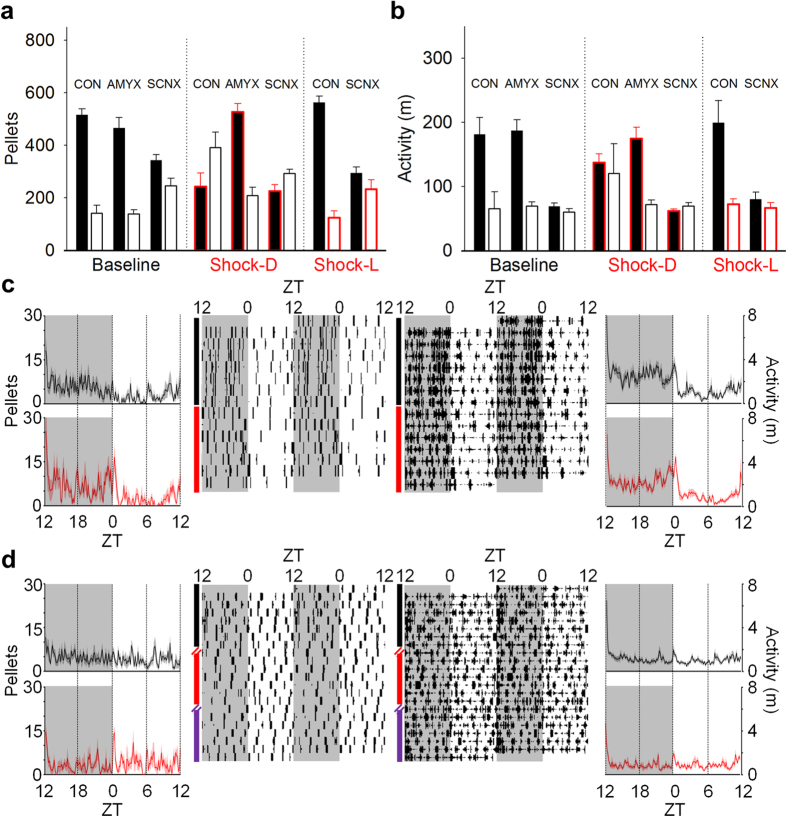
Effects of lesions on shock-induced changes to circadian rhythms. (**a**) Mean number of pellets and (**b**) mean activity (distanced travelled in m) in the dark (*filled bars*) and light (*open bars*) phases of AMYX (n = 7), SCNX (n = 8), and sham (CON; n = 7) animals during the last 5 d of baseline (*left*), unsignaled nocturnal (*center*) and unsignaled diurnal shock (*right*; SCNX and CON only). Red highlights indicate shock period. Error bars represent SEM. (**c**) 24-h waveforms (*outside*) and raster plots (*inside*) of feeding (*left*) and activity (*right*) from amygdala-lesioned rats (n = 7; baseline and unsignaled shock conditions represented by *black* and *red*, respectively). (**d**) 24-h waveforms (*outside*) and raster plots (*inside*) of feeding (*left*) and activity (*right*) from SCN-lesioned animals (n = 8). Waveforms show the mean feeding and activity over 24 h (bold lines), in 10-min time-bins, of all animals in each group averaged over the last 5 d of each experimental condition. SEM is shown as the shaded areas above and below the bold lines.

In order to examine whether these changes in feeding and activity were dependent on known circadian timing- and fear-related brain structures, the effects of unsignaled nocturnal footshocks on circadian feeding and activity were examined in SCN- or amygdala-lesioned rats (Fig. 6). During baseline, amygdala-lesioned (AMYX; Fig. 7a–d) rats were significantly biased toward the dark phase, as measured by feeding [dark: 454.69 ± 41.02 pellets, light: 168.27 ± 29.58 pellets] (Fig. 7a,b) and activity [dark: 193.81 ± 11.20 m; light: 72.63 ± 4.64 m] (Fig. 7c,d). This dark phase preference did not change when unsignaled nocturnal shocks were presented [feeding: condition × phase, *F*_1, 6_ = 1.98, *p* = 0.21; activity: condition × phase, *F*_1, 6_ = 0.001, *p* = 0.98], and feeding even increased overall during the unsignaled shock condition [*F*_1, 6_ = 7.38, *p* = 0.035]. On the other hand, while arrhythmic SCN-lesioned (SCNX; Fig. 7e–h) rats were still slightly biased toward the dark phase in feeding [dark: 353.86 ± 22.14 pellets, light: 281.93 ± 27.05 pellets; *F*_1, 7_ = 20.49, *p* = 0.003] (Fig. 7e,f) and activity [dark: 80.51 ± 6.50 m, light: 60.02 ± 6.90 m; *F*_1, 7_ = 32.49, *p* = 0.001] (Fig. 7g,h) during baseline the introduction of unsignaled nocturnal shock abolished this dark phase preference for feeding [*F*_1, 7_ = 4.26, *p* = 0.078]. Activity remained slightly biased toward the dark phase [*F*_1, 6_ = 12.74, *p* = 0.012], though dark phase activity decreased over time [phase × day: *F*_13, 78_ = 4.26, *p* < 0.001]. Because SCNX rats still showed, to some extent, greater nocturnal activity and feeding during baseline, unsignaled shock was then switched to occur only during the light phase to examine whether this would augment their dark phase bias. Despite diurnal unsignaled shock, both feeding [phase: *F*_1, 7_ = 0.43, *p* = 0.53] and activity remained arrhythmic [phase: *F*_1, 7_ = 2.63, *p* = 0.15]. The mean feeding and activity behavior for each group are summarized in Fig. 8a,b, respectively. Unlike intact animals, neither AMYX nor SCNX rats showed anticipatory feeding [AMYX: condition × time, *F*_23, 92_ = 1.339, *p* = 0.165; SCNX: *F*_23, 92_ = 0.940, *p* = 0.548] or activity [AMYX: condition × time, *F*_23, 92_ = 0.746, *p* = 0.786; SCNX: *F*_23, 92_ = 1.131, *p* = 0.329] before the LD transition (Fig. 8c,d). These results indicate that both the SCN and amygdala are necessary to shift circadian foraging rhythms away from the time of threat and generate anticipatory activity organized around safe periods.

## Discussion

The significance of environmental threats to daily behavior is apparent from naturalistic studies that have reported that increased predation risk or hunting by humans is associated with profound changes in the activity patterns of mammalian prey^8^,^11^,^28^,^37^. For instance, one study^7^ observed a population of wild rats (*Rattus norvegicus*) exhibiting diurnal activity that shared a habitat with red foxes, a nocturnal predator. After a subset of the diurnal rats were captured and kept in a safe enclosure, they reverted to being nocturnal, and it was concluded that the rats were diurnal in order to avoid predation^7^. Another study examining the temporal and spatial activity of wild boar (*Sus scrofa* L.) as a function of hunting pressure found that areas with increased diurnal hunting by humans were associated with a reduction in the boars’ diurnal activity^38^. However, it has remained unclear whether such shifts in activity represent simple conditioned fear responses to predatory stimuli or reprogramming of circadian rhythms. The present study employed an ethologically-relevant foraging setting that simulates the environment in which circadian rhythms likely evolved and demonstrates that time-specific fear can serve as a non-photic zeitgeber entraining a circadian oscillator that times foraging behavior to a non-threatening time of the day. The discovery that a dark phase-associated unsignaled threat leads to endogenous persistent rhythmic activity just before and during the safe light period provides further evidence against a strictly Pavlovian interpretation in which the LD cycles as a conditioned stimulus.

Previous studies using hamsters and Wistar rats have reported that the strength of conditioned place preference (CPP) and avoidance (CPA) is modulated by the time of day of training^39^,^40^. That is, the expression of the CPP or CPA was strongest when tested 24 and 48 h after training but was not exhibited at 32 or 40 h intervals. These effects were present even with lesions to the SCN, suggesting the mechanism of this “time-stamped” learning was not dependent on circadian oscillators in the SCN^41^. It is important to note that the effects observed in the CPP experiments may have involved food-entrainable oscillators as the animals were food restricted and also that Long-Evans rats, unlike Wistar rats, did not exhibit time-dependent expression of CPA^40^,^41^. Our experiments used Long-Evans rats that were not food-restricted, and the anticipatory behavior we observed depended on an intact SCN. Thus, the effects were likely due to an SCN-dependent mechanism rather than the time-stamped learning suggested by Ralph and colleagues^41^. These shifts in circadian feeding and activity may also be context-dependent, as other studies have reported a lack of phase-shifting following exposure to stressors presented in a different context than where circadian behavior was measured^25^,^42^.

The present effect is also distinct from proposed “cognitive oscillators” that shift behavior toward periods requiring heightened attention^20^ in that the fear-induced oscillator shifts behavior *away* from the time associated with unpredictable threat, which may represent a period requiring greater attentional demand. Furthermore, the dependence of this fear-induced oscillator on an intact amygdala indicates that the circadian neural network that temporally orchestrates complex behaviors likely includes limbic centers that encode aversive stimuli and coordinate fear and anxiety responses. It is important to note that, while there is some debate regarding the use of the term “fear” with regard to non-human animals^31^,^43^, we use “fear” here to refer to the central defensive-motivational state activated by aversive stimuli^27^,^29^,^44^,^45^.

Similarly to the food-entrainable oscillator, which times activity to a restricted feeding schedule, the fear-entrainable oscillator we describe overrides entrainment of rhythmic foraging and activity by the LD cycle^13^. However, unlike the food-entrainable oscillator, which does not depend on an intact SCN, the fear-entrainable oscillator depends both on an intact SCN and intact amygdala. Thus, the fear-entrainable oscillator could reside within the SCN itself or, alternatively, it could be an extra-SCN oscillator to which the SCN relays information on the phase of the LD cycle. Alternatively, cyclic fear could modulate the phase relationship between the SCN pacemaker and downstream oscillators that time behavior. However, unless the change in this phase relationship involves the entrainment of a circadian oscillator, the original phase relationship should be restored upon removal of the cyclic fear, which was not the result we obtained. Finally, the cyclic fear stimulus could change the phase of entrainment of the SCN to the LD cycle, but we do not favor this interpretation. Although it has been reported that an aversive stimulus can inhibit photic phase-shifting when paired with light pulses^26^, we are unaware of cyclic non-photic stimuli that can change the steady-state phase of entrainment of the master circadian clock to the LD cycle.

If the shift in feeding and locomotor activity involves an anatomically identifiable fear-entrainable clock, what could be the site of this oscillator? The central and basolateral nuclei of the amygdala exhibit 24-h rhythmic expression of *Per2* that is apparently dependent on intact SCN oscillations^46^,^47^. Clock genes are not only critical components of the transcription-translation feedback loop that constitutes circadian oscillators but they are also part of complex gene networks that transduce physiological and behavioral conditions including metabolic, nutritional and emotional state^48^. It is therefore plausible that an oscillator in the amygdala that is sensitive to threatening stimuli works synergistically with the SCN to gate behavior to a time of the LD cycle when the threat of harm or predation is not present. Indeed, direct projections from the SCN to the central amygdala have been shown^49^,^50^, but the function of this projection remains unknown. Similarly, the dorsomedial nucleus of the hypothalamus and the SCN have been proposed to participate in a multi-oscillatory system to regulate food anticipatory activity after restricted feeding schedules^51^.

It remains to be determined whether cyclic threat entrainment of foraging and feeding behaviors also involves reprograming of other circadian rhythms. For instance, could fear-entrained oscillator(s) change the timing of endocrine rhythms, such as the evening peak of glucocorticoid release in nocturnal rodents^52^? Recent studies have shown that when mice have to “work” to obtain food they switch to a diurnal pattern of activity, and this switch can be explained by the beneficial effects that the diurnal pattern of activity has, as it lowers energy expenditure^53^,^54^. This work-for-food switch in the activity pattern is associated with changes in the daily release of corticosterone as well as the phase of some peripheral circadian oscillators. Future studies could determine the extent of the circadian reprograming that can be induced by cyclic threat.

In summary, the present study found that amygdala-coded fear can reprogram circadian behavior to override behavioral outputs of LD-entrained oscillators. Our observations suggest an intriguing possibility that the amygdala and the SCN interact as a fear-entrained oscillator to adapt to cyclic predatory threats by predicting times of danger and safety and organizing circadian behaviors accordingly. Further research on cellular and molecular mechanisms underlying this circadian reprograming may illuminate new avenues for treating people with anxiety-related disorders, such as posttraumatic stress disorder, that disrupt normal daily rhythms.

## Methods

### Animals and Apparatus

Naïve, male Charles River Long-Evans rats initially weighing 275–300 g were individually housed in eight closed economy chambers (Fig. 1a) on a 12 h/12 h LD cycle^35^. The closed economy dimensions were 74.3 cm × 25.4 cm × 33 cm (length × width × height) and consisted of a ‘nest’ (20.3 × 25.4 cm) and a ‘foraging’ arena (54 × 25.4 cm). The nest floor was covered with sawdust, while the floor of the foraging arena was composed of 32 stainless steel rods (4.5 mm diameter) wired to a precision animal shocker (Coulbourn Instruments, Allentown, PA) for delivery of footshocks. A camera (Fire-I B/W Board camera; Unibrain Inc., San Ramon, CA) was mounted above each closed economy chamber and connected to a computer for tracking animal activity via ANY-maze software (Stoelting, Wood Dale, IL), which also measured activation of the food levers and dispensers (Med Associates, Inc., Georgia, VT) and the shock generator connected to an ANY-maze Interface (AMi; Stoelting, Wood Dale, IL). Forty five-mg grain-based pellets for rodents were used in all experiments (#F0165, Bio-Serv, Flemington, NJ). The total number of pellets dispensed was chosen as the primary *foraging* variable due to the necessity of the animal to be in the foraging area and actively pressing the lever in order to obtain and consume the food pellets. Importantly, all pellets obtained by each animal were consumed. The total distanced traveled (m) among both the foraging and nest areas, rather than within one specific area, was used as the primary *activity* variable for analyzing circadian rhythms as this would include but not be confounded by avoidance behavior. White noise (70 dB) generated by the ANY-maze software was continuously played through computer speakers throughout the experiment to obscure external noises. The actual times corresponding to the onset of the dark and shock phases (ZT12) occurred between 10 a.m. and 3 p.m. (varied by cohort) so any human work-related vibrations would be poorly associated with the experimental cycles.

All animal experiments were conducted in compliance with the National Institutes of Health Guide for Care and Use of Laboratory Animals and were reviewed and approved by the University of Washington Institutional Animal Care and Use Committee.

### Procedures

After arrival, animals were given 10–14 d to acclimatize to the chamber (e.g., lever press for food pellet on a continuous reinforcement schedule) and entrain to the LD cycle (as confirmed by an actogram). After 7 baseline days, half of the animals were exposed to 14 d of unsignaled, pseudo-random footshocks (0.8 mA; ~2 shocks/h in the dark phase only) followed by 14 d of signaled, pseudo-random footshocks (9-s light cue preceding the shock in the dark phase only). This was counterbalanced by the other half of the animals (assignment randomized; Fig. 1b). If the animal was in the foraging area when ANY-maze triggered the footshock, the shock continued for up to 10 s or until the animal escaped to the nest. In the signaled footshock condition, if the animal moved to the nest within 9 s of a light cue (i.e., avoidance response), then the light (Med Associates, Inc., Georgia, VT) promptly terminated and no shock occurred. If the animal failed to enter the nest within 9 s of the light, the footshock ensued and both stayed on for up to 10 s or until the animal escaped to the nest. If the animal was in the nest at the time the program generated the light or the footshock, they both terminated instantaneously. Since only the unsignaled nocturnal footshocks caused animals to switch the majority of their feeding behavior to the light phase, a signaled footshock condition was not included in the free-running or lesion experiments.

Animals were removed from the closed economy chambers during the last hour of the light phase (ZT11-ZT12) every 2–3 days so the chambers could be cleaned, the food and water replenished, and animals weighed. Animals were returned to the experimental chambers at the beginning of the dark phase (ZT12). This was the extent of experimenter-animal interaction. In the free-running experiment (Fig. 1c), animals were undisturbed for 3 d to assess free-running under constant dark conditions. In the lesion experiment, animals were only exposed to 14 d of unsignaled nocturnal shock, except for the SCN lesion and sham animals, which experienced an additional 14 d of diurnal (light phase only) shock to further examine the SCN lesion effect on arrhythmia.

### Surgery

Under anesthesia (30 mg/kg ketamine and 2.5 mg/kg xylazine, i.p.), rats were randomly assigned to receive either bilateral electrolytic lesions to their amygdalae (AMYX group, n = 7; from bregma: AP −2.5; ML +4.2/5.0; DV −8.4/8.6 mm)^55^, the SCN (SCNX group, n = 8; from bregma: AP −1.3; ML + 0.3; DV −9.1)^55^ or had lesion electrodes inserted 1 mm above the amygdala or the SCN, except current was not delivered (CON, n = 7; sham groups combined because there were no statistical differences). For the AMYX group, lesions were made by passing constant current at 1.0 mA for 10 s (Grass Medical Instrument, Quincy, MA) through epoxy-coated insect pins (#00, ~0.75 mm tip exposed)^35^. For the SCNX group, lesions were made by passing constant current at 1.75 mA for 17.5 s (Grass Medical Instrument, Quincy, MA) through epoxy-coated insect pins (#00, ~.25 mm tip exposed)^56^.

### Histology

At the completion of the experiment, all rats were overdosed with Beuthanasia and perfused intracardially with 0.9% saline followed by 10% buffered formalin. The brains were removed and stored in 10% formalin overnight and then kept in 30% sucrose solution until they sank. Transverse sections (50 μm) through amygdalar and SCN lesions were taken, mounted on gelatin-coated slides, and stained with cresyl violet and Prussian blue dyes for confirmation of electrode placement and lesion accuracy.

### Statistical Analysis

All data are presented as mean ± SEM. Group sizes were selected based on power analyses performed using G*Power 3.1 software with estimates of effect sizes obtained from previously-published findings in the lab (Kim *et al.*, 2014). The behavioral data (with the exception of the free-running experiment, see below) was analyzed using mixed factorial ANOVAs on the daily total number of pellets dispensed and total distance traveled (in m) with the within-subject factors of time (day or 10-min time-bin), light phase, and experimental condition (baseline, signaled, or unsignaled shock). In cases where the assumption of sphericity was violated (Mauchly’s test), Greenhouse-Geisser corrected degrees of freedom were used. In cases where Levene’s Test for Equality of Variance was significant, the degrees of freedom were corrected using the Welch-Satterthwaite method. Bonferonni-adjusted, two-tailed, paired-samples t-tests or independent t-tests were used for post hoc tests where appropriate. Free running rhythms were analyzed first by visual inspection of actograms, then a Sokolov-Bushel periodogram on the days of free-running was used to determine the circadian period of each animal. The period for each animal was then used to construct waveforms of the successive days of feeding and activity. Each of these two waveforms was analyzed by a one-way ANOVA to determine a significant effect of time^51^. Two animals did not show significant periods for feeding, and were thus not included in the mean feeding free-running analysis. All animals showed significant periods for the measure of activity. Time-series analysis was done with ElTemps software (A. Díez Noguera, University of Barcelona, Spain). Statistics were performed using SPSS (version 18.0). Five animals were excluded from the analysis due to incomplete, misplaced, or unilateral lesions to the amygdala (four) or SCN (one), and one CON animal was excluded due to health complications following surgery. Three animals were excluded for failing to entrain to the LD cycle during the initial acclimation period.

## Additional Information

**How to cite this article**: Pellman, B. A. *et al.* Time-Specific Fear Acts as a Non-Photic Entraining Stimulus of Circadian Rhythms in Rats. *Sci. Rep.* **5**, 14916; doi: 10.1038/srep14916 (2015).
